# Distinct skin microbiome signatures in Black and White children with food allergy and asthma

**DOI:** 10.1111/pai.70197

**Published:** 2025-09-04

**Authors:** John P. Fyolek, Mahboobeh Mahdavinia, Jialing Jiang, Lucy A. Bilaver, Susan Fox, Sai R. Nimmagadda, Pamela J. Newmark, Hemant Sharma, Amal Assa'ad, Patrick C. Seed, Ruchi S. Gupta, Christopher M. Warren

**Affiliations:** ^1^ Center for Food Allergy and Asthma Research and Institute for Public Health and Medicine Northwestern University Feinberg School of Medicine Chicago Illinois USA; ^2^ Division of Allergy and Immunology, Department of Medicine and Department of Pediatrics University of Texas Health Sciences at Houston Houston Texas USA; ^3^ Division of Allergy and Immunology, Department of Medicine and Department of Pediatrics Rush University Medical Center Chicago Illinois USA; ^4^ Ann & Robert H. Lurie Children's Hospital of Chicago Chicago Illinois USA; ^5^ Division of Allergy and Immunology Children's National Health Systems Washington District of Columbia USA; ^6^ Division of Allergy and Immunology, Cincinnati Children's Hospital Medical Center The University of Cincinnati Cincinnati Ohio USA; ^7^ Division of Infectious Disease Ann & Robert H. Lurie Children's Hospital of Chicago Chicago Illinois USA

**Keywords:** asthma, food allergy, microbiome, skin

To the Editor,

The prevalence of food allergy (FA) and other atopic conditions has risen over the last several decades.^1^ Many recent studies point out marked disparities in outcomes of atopic diseases impacting historically underrepresented populations. Most atopic diseases are more prevalent and severe in Black individuals compared to White individuals.^2^, ^3^ Black children in the US are more likely to suffer from adverse events related to FA and have a higher risk of developing other atopic comorbidities.^2^

A potential explanation for these trends could be that environmental exposures in modernized society alter the composition and diversity of the human microbiome and contribute to FA and atopy development.^4^ The resulting microbial dysbiosis impacts the immune system through inflammatory dysregulation, which consequently predisposes the individual to atopic diseases.^4^ It is noteworthy that the relative abundance of several gut bacteria is significantly different in Black children compared to White children with FA, and the microbial composition in Black children with FA is found to be indicative of asthma.^5^

Food allergen profiles differ between Black and White children in the US.^2^ Some of these differences in food allergen profiles have been shown to be mediated by differential exposure and sensitization to specific cross‐reactive components, such as dust mite and cockroach.^6^, ^7^ In addition to the gut microbiome, the skin microbiome may also play a role in FA presentation. The skin acts as a key barrier to the external exposome, and epithelial function can be impacted by changes in skin microbiota^8^, ^9^ which is heavily driven by environmental exposures.^10^ However, the relationship between the skin microbiome and atopic disease pathogenesis, as it relates to ethnic differences in FA outcomes, is unclear.

Skin samples of 256 children between 3 and 12 years old were obtained and analyzed to identify differences in the microbial composition of Black and White children with FA, and whether differences were associated with the presence of comorbid asthma. Samples were obtained from subjects in the FORWARD study with informed consent as previously described, and research activities were IRB approved.^6^ Enrolled Black and White children had similar age and gender distribution. There were no significant differences in alpha diversity in either the Chao1 or Shannon indices, or the overall bioburden of bacteria between Black and White groups. Microbial composition appeared similar across Black and White subjects, and PERMANOVA analysis confirmed the difference in microbial composition between the two groups was not significant (*R* ^2^ = 0.005, *p* = .068). Spearman correlation analysis of the Shannon index indicated that the evenness in microbial diversity increases with age (*R* = 0.11, *p* = .043). Shannon diversity was attenuated in a generalized linear model controlling for age, indicating that age has significant (*p* < .001) influence on Shannon diversity of the skin microbiota. Microbial community composition was not significantly linked to age (*R* ^2^ = 0.05, *p* = .09) (Figure S1). These results are consistent with what was observed in gut microbiota in early life of children with FA, which indicates that, like gut microbiota, age is not a major determinant of skin microbial composition in children >3 years of age.^5^, ^11^

Differentially abundant taxa between Black and White children were determined by beta binomial regression while adjusting for the child's age and area deprivation index (ADI) scores. ADI was used as an encompassing measure to control for socioeconomic disadvantages. Other potential confounders such as antibiotic use and family history of atopy were tested in the model but did not significantly alter model performance and identification of differentially abundant taxa; therefore, they were not included in the final model to maintain clarity and efficiency. Several taxa were found to be differentially abundant between Black and White children (Figure 1). *Acinetobacter* (8 species), *Corynebacterium* (6 species) and *Sphingomonas* (4 species) were the most common genera reduced in Black children, while, in contrast, the relative abundance of *Psychrobacter* (8 species) was increased.

**FIGURE 1:**
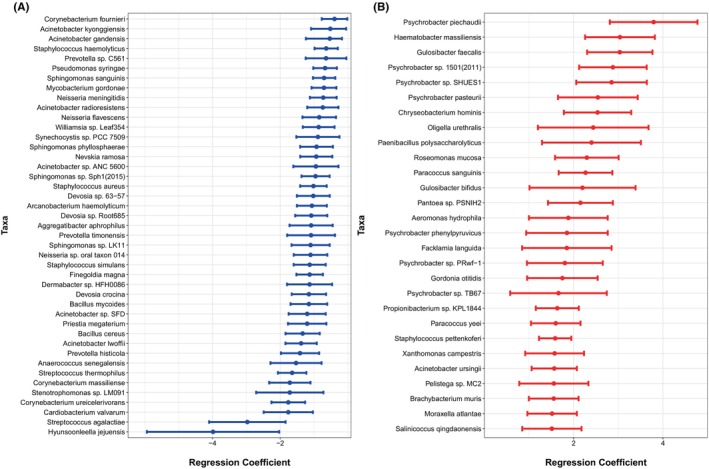
Differentially abundant taxa in black vs. white subjects controlling for age. FDR <0.05 is significant. (A) Taxa with a decreased abundance in black compared to white subjects. (B) Taxa with an increased abundance in black compared to white subjects.

Comorbid asthma was significantly more prevalent in Black participants (*p* = .02) (Table 1). Asthma was associated with the differential abundance of several species in Black subjects, including reduced relative abundance of 4 *Corynebacterium* and 2 *Acinetobacter* species (Figure 2). Next, the differences in children with asthma were assessed while adjusting for the child's age and ADI. Twenty‐three differentially abundant taxa were identified. Notably, Black children with asthma and FA had a lower abundance of *Corynebacterium ureicelerivorans, Corynebacterium afermentans, Corynebacterium jeddahense*, *Acinetobacter baumannii*, and *Acinetobacter parvus* when compared to White children with asthma and FA.

**TABLE 1:** Demographics and clinical data of 256 children (3–13 years Old at the time of enrollment) with Ige‐mediated FA(S) enrolled into FORWARD (Food Allergy Outcomes Related to White and African American Racial Differences) microbiota study.

	Black or African American	White
*N*	92	164
Age (years)		
3–5 (%)	13%	23%
5–8 (%)	32%	30%
8–10 (%)	17%	18%
10–12 (%)	38%	29%
Mean ± SD (years)	8.44 ± 2.83	7.27 ± 2.84
Gender		
Male (%)	58%	60%
Atopic Comorbidities		
Asthma	**60 (65.2%)**	**59 (36%)**
Eczema	76 (82.6%)	135 (82.3%)
Age of Comorbidities (Mean ± SD, years)		
Asthma	9.09 ± 2.55	7.82 ± 2.63
Eczema	8.29 ± 2.86	7.28 ± 2.82

*Note*: Significantly different fields are bold.

**FIGURE 2:**
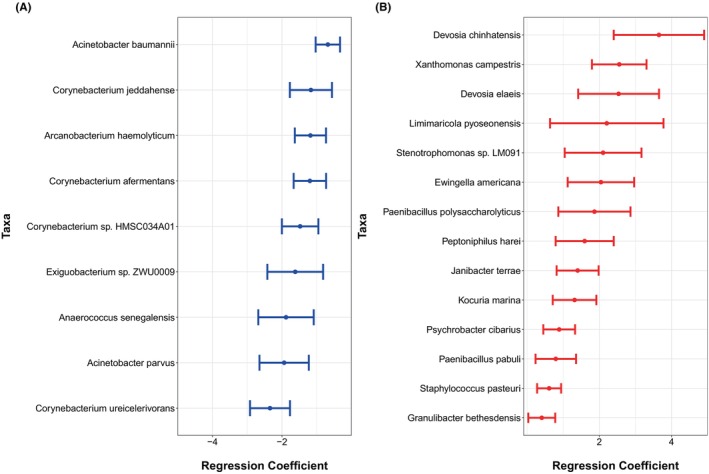
Differentially abundant taxa in subjects with asthma controlling for age and race. FDR <0.05 is significant. Taxa with a decreased abundance (A) and increased abundance (B) in black subjects with asthma compared to white subjects with asthma.

Important groups of bacteria that were significantly decreased on the skin of Black children compared to their White counterparts belonged to *Acinetobacter* and *Corynebacterium* genera. A sparse compositional mediation model adjusted for age and ADI was performed to evaluate the effects of differentially abundant *Corynebacterium* and *Acinetobacter* species in asthma subjects with food allergies between Black and White subjects. The direct effect was significant (*p* < .001) with an estimated coefficient of −0.183 (95% CI; −0.223, −0.144), indicating an inverse relationship with asthma in Black subjects with FA. Both *Acinetobacter* and *Corynebacterium* are diverse genera and dominant members of the human skin microbiome.^12^, ^13^, ^14^ Importantly, several species belonging to these two genera were less abundant in children with asthma. *Corynebacterium* is previously shown to be protective against allergic sensitization.^15^ *Acinetobacter* has also been associated with protection from atopic conditions in other cohorts.^12^ A previous study comparing two European populations showed a decreased abundance of several species of *Acinetobacter* on skin linked to increased incidence and severity of multiple atopic conditions including asthma and allergic rhinitis.^12^ This epidemiological finding is backed up by mechanistic studies showing that the *Acinetobacter* species on skin regulate the expression of anti‐inflammatory molecules by PBMCs.^14^, ^16^ The balance between TH1/TH2 gene expression and the anti‐inflammatory molecules was related to the composition of the skin microbiota, specifically the abundance of *Acinetobacter*.^16^ In a mouse model, *Acinetobacter* species induced a TH1‐skewed inflammatory process, along with anti‐inflammatory responses by immune cells and skin cells that protected against allergic sensitization and lung inflammation through the skin.^14^, ^16^ Gatekeeping bacteria on human body surfaces are key elements of the immune system response, and their imbalance in Black children is concerning. The decreased RA of gatekeeping *Acinetobacter* species in Black children might be at least partially responsible for the observed higher rate of asthma in Black children with FA.^2^, ^17^

Several *Psychrobacter* species were found to be relatively increased on the skin of Black children compared to White children in our cohort. These bacteria are aerobic, Gram‐negative, and psychrophilic, and while they are not commonly associated with disease in the general population, they have been identified in rare cases of opportunistic infections, particularly in immunocompromised individuals.^18^ On the skin, *Psychrobacter* has been observed in association with certain dermatological conditions, such as seborrheic dermatitis and psoriasis, although its exact role remains unclear.^19^ The increased presence of *Psychrobacter* may not inherently indicate pathogenicity; rather, its impact could depend on its interactions with other microbial species and the host immune environment.

Multiple *Staphylococca*l species including *Staphylococcus aureus* (*S. aureus*) were decreased on the skin of Black children. *S. aureus* colonization on the skin has been positively associated with the presence and severity of eczema.^20^ *S. aureus* enterotoxins have been linked to the exacerbation of asthma symptoms and are known to trigger elevated levels of IL‐5 and IgE.^21^ Although this relative decrease may appear beneficial, the higher prevalence of asthma and the comparable rates of eczema between Black and White children in this cohort suggest a more complex interaction. It may not be the mere presence or abundance of *S. aureus* that is most relevant, but rather the amount of toxin it produces or its relative abundance in relation to other gatekeeping microbial species. These factors could influence host immune responses and disease expression in ways that are not captured by abundance alone.

In our cohort, the skin of Black children with food allergy showed a relative increase in several bacterial taxa, including *Gulosibacter faecalis*, *Haematobacter*, *Oligella*, and *Chryseobacterium hominis*. While these species are not traditionally dominant members of the skin microbiome, their increased presence may reflect broader ecological shifts influenced by host genetics, immune responses, or environmental exposures. The functional roles of these bacteria on the skin remain largely unexplored, but their abundance could indicate altered microbial interactions, potential displacement of protective commensals, or changes in immune modulation. These findings highlight the importance of considering microbial community structure and function—not just individual taxa—in understanding the skin microbiome's role in allergic disease expression.

Other bacteria that were significantly decreased in Black children with FA were species of *Sphingomonas*, which act as important skin commensal bacteria with a role in β‐galactosidase activation and inhibition of the expression of cell cycle inhibitors.^22^ Furthermore, these bacteria have a significant positive impact on epidermal restructuring and delay intrinsic skin senescence.^22^ It is important to investigate whether the decreased abundance of *Sphingomonas* species in Black children with FA impacts the skin barrier.

Human activities in the urban environment have led to simplified urban bacterial diversity, which results in reduced microbial exposure.^23^ Environmental exposure to soil is closely linked to the skin microbiota.^24^ The reduced abundance of commensal gatekeeping bacteria in Black children's skin might be due to their higher exposure to urban materials and increased exposure to hazardous environmental factors in impoverished urban neighborhoods with limited greenspace and more pollution.^25^ Several of above‐discussed bacteria, including *Actinobacteria* and *Sphingomonas*, are impacted by environmental parameters.^26^ ADI scores were markedly different in Black and white children; however, ADI was not a significant factor in the abundance of any species belonging to the *Corynebacterium*, *Acinetobacter*, or *Sphingomonas* genera. While our finding of differential skin microbiota and depletion of gatekeeping bacteria in Black children with FA and its link to asthma is potentially concerning, there is recent research showing that this phenomenon might be preventable and potentially reversed.^24^ Further research in this area is needed to expand our knowledge and provide solutions.

## AUTHOR CONTRIBUTIONS

**John P. Fyolek:** Conceptualization; investigation; methodology; visualization; writing – review and editing; formal analysis; writing – original draft; data curation. **Mahboobeh Mahdavinia:** Conceptualization; investigation; funding acquisition; writing – review and editing; resources; supervision; formal analysis. **Jialing Jiang:** Writing – review and editing; data curation; resources; investigation; project administration. **Lucy A. Bilaver:** Funding acquisition; writing – review and editing; supervision; data curation; resources; conceptualization. **Susan Fox:** Project administration; writing – review and editing; investigation. **Sai R. Nimmagadda:** Funding acquisition; supervision; resources; project administration; writing – review and editing; investigation. **Pamela J. Newmark:** Project administration; data curation; supervision; resources; writing – review and editing. **Hemant Sharma:** Writing – review and editing; project administration; supervision; resources; investigation. **Amal Assa'ad:** Investigation; writing – review and editing; project administration; supervision; resources. **Patrick C. Seed:** Conceptualization; methodology; writing – review and editing; data curation; supervision; resources. **Ruchi S. Gupta:** Conceptualization; investigation; funding acquisition; project administration; supervision; resources. **Christopher M. Warren:** Conceptualization; investigation; funding acquisition; writing – review and editing; project administration; supervision; resources; formal analysis.

## FUNDING INFORMATION

This study was supported by the National Institute of Health (Grant Number: 1R01AI130348‐01A1).

## CONFLICT OF INTEREST STATEMENT

Dr. Gupta, Mahdavinia, Warren, Bilaver, and Assa'ad report other research support from the National Institutes of Health and Food Allergy Research & Education (FARE). Dr. Bilaver receives research grant support from Rho, Genentech, National Confectioners Association, Novartis, and Before Brands Inc. Dr. Warren reports other research support from the Sunshine Charitable Foundation. He reports receiving honoraria for the development of CME review articles on epidemiology from the American College of Allergy, Asthma, and Immunology and the American Academy of Allergy, Asthma, and Immunology. The authors whose names are listed certify that they have no other affiliations with or involvement in any organization or entity with any financial interest (such as honoraria; educational grants; participation in speakers' bureaus; membership, employment, consultancies, stock ownership, or other equity interest; and expert testimony or patent‐licensing arrangements), or non‐financial interest (such as personal or professional relationships, affiliations, knowledge or beliefs) in the subject matter or materials discussed in this manuscript. Furthermore, listed authors testify on behalf of all members of the FORWARD group that they have no conflict of interest associated with this publication.

## PEER REVIEW

The peer review history for this article is available at https://www.webofscience.com/api/gateway/wos/peer‐review/10.1111/pai.70197.

## Supporting information


Appendix S1.
