# Supplementary material for: Distinct skin microbiome signatures in Black and White children with food allergy and asthma
Source: Pediatr Allergy Immunol. 2025 Sep 4;36(9):e70197. doi: 10.1111/pai.70197 (PMC12410127; doi:10.1111/pai.70197)

**SUPPLEMENTARY MATERIAL**

**S1**

**Methodology**

Children younger than 12 years old with allergist-diagnosed IgE-mediated FA were enrolled from four centers across the USA; Ann & Robers H. Lurie Children’s Hospital of Chicago (Chicago, IL), Rush University Medical Center (Chicago, IL), Children’s National Hospital (Washington, D.C.) and Cincinnatti Children’s Hospital Medical Center (Cincinnatti, OH). Skin microbiota samples were collected in clinic from retro-auricular creases and antecubital fossa using Catch-All^TM^ Sample Collection Swab and immediately transferred to the lab to be stored in -80^o^C freezer. Skin samples were only collected from participants who had not taken systemic antibiotics within the two months prior to sampling. Additionally, no topical antibiotics or corticosteroids were used on the sampled skin sites for at least two months prior to collection. These criteria were implemented to minimize potential confounding effects of recent medication use on the skin microbiome and to ensure the integrity of the microbial profiles analyzed.

We followed established protocols for handling low-biomass skin microbiome samples, which included careful sample collection using sterile swabs, gloves, and clean environments to minimize contamination. DNA was extracted from thawed swab and scrape samples using the QIAamp DNA Microbiome Kit – Swab and Body Fluid DNA Isolation (Qiagen), specifically designed for low-biomass samples such as skin swabs. This kit incorporates differential lysis and enzymatic digestion to effectively deplete host DNA, along with mechanical and chemical lysis for efficient bacterial cell disruption. Ultra-clean spin columns further reduce the risk of contamination. The extracted DNA is suitable for whole metagenome shotgun sequencing. All samples underwent bead beating using the Qiagen TissueLyser at maximum frequency for 10 minutes following incubation. DNA was eluted in 50 µl of elution buffer and stored at –20°C until further processing. To monitor contamination and ensure data quality, negative controls (PCR-grade water without skin samples) and a positive control mock community were processed in parallel with the study samples. Potential contaminant sequences were identified and removed using the Decontam R package, which detects contaminants based on their prevalence in negative controls relative to true samples. Shotgun metagenomic sequencing was used to comprehensively profile microbial communities. DNA was sequenced using a 150 base paired-end protocol on the Illumina HiSeq 4000. High-quality filtered sequences were clustered into reference gene catalogs. Functional annotation of the catalogs was performed using the eggNOG database. Additional gene content information from 18 gene function databases was obtained by using available eggNOG-to-database alignments. Taxonomic profiles were generated by mapping reads to the mOTU and RefMG marker gene catalogs. Gene length normalized counts were computed for subsequent analysis. Bacterial genome copies per sample (skin swab) were measured by qPCR to indicate the overall bioburden of the measured microbiome niche using an established TaqMan assay. The differences in taxonomic composition between groups was evaluated using Bray-Curtis distances and statistical differences between communities was assessed using PERMANOVA controlling for covariates. Wilcoxon rank-sum tests were used to evaluate alpha diversity. Differences in relative abundance were determined using beta binomial regression with R package corncob (v. 0.3.1), controlling for age, gender, and other comorbid factors as indicated. Analyses were corrected for false discovery rate (FDR) using Benjamini-Hochberg procedure. These analyses were completed using R (4.2.1)


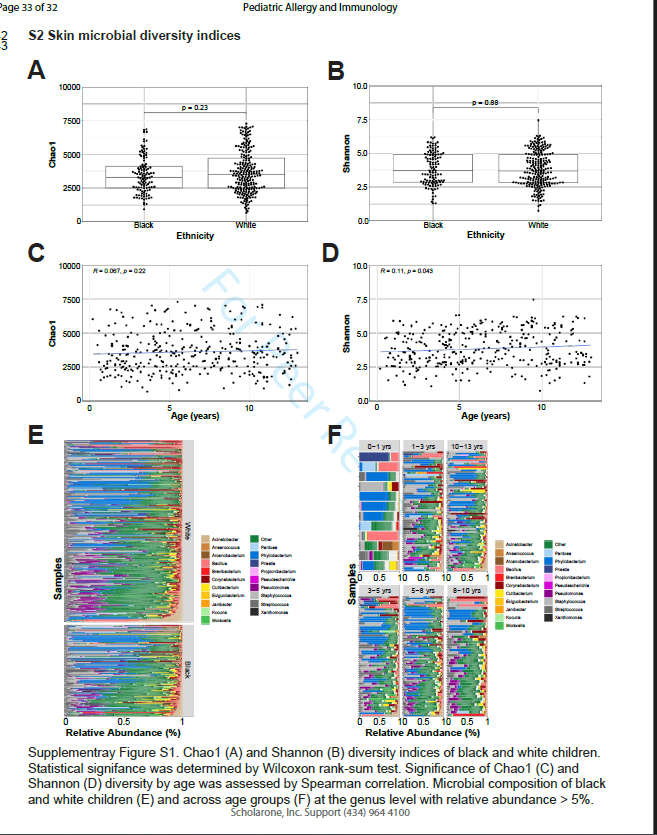

Supplement: Supplementary file 1 — Appendix S1. [file PAI-36-e70197-s001.docx]
